# Response to Letter to the Editor: “Changes in Thyroid Function Across Adolescence: A Longitudinal Study”

**DOI:** 10.1210/clinem/dgaa315

**Published:** 2020-05-27

**Authors:** Purdey J Campbell, Phillip Kendrew, Michelle Lewer, Ee Mun Lim, Scott G Wilson, John P Walsh

**Affiliations:** 1 Department of Endocrinology & Diabetes, Sir Charles Gai rdner Hospital, Nedlands, WA, Australia; 2 Pathwest Laboratory Medicine, Nedlands, WA, Australia; 3 School of Biomedical Sciences, University of Western Australia, Crawley, WA, Australia; 4 Department of Twin Research & Genetic Epidemiology, King’s College London WC2R 2LS, London, UK; 5 Medical School, University of Western Australia, Crawley, WA, Australia

We thank Dr. Raverot and colleagues for their comments, which highlight the challenges and complexities of establishing appropriate reference intervals. With regard to assay performance, inter-assay coefficients of variation in our study were as follows: TSH, 3.3% and 3.2% at 0.37 and 4.3 mU/L respectively; FT4, 4.8% and 5.9% at 10 and 28 pmol/L, respectively; and FT3, 4.8% and 3.4% at 3.7 and 9.9 pmol/L, respectively. We confirm that the study was performed before the change from 2-point to 6-point calibration for FT3. We are aware of the data regarding biological variation in TSH, FT4, and FT3, but these would not account for the longitudinal trends and sex difference in FT3 shown in Figure 3 of the paper, nor is it relevant to determining reference intervals.

We agree that achieving harmonization of thyroid function tests is a worthy goal, but challenges will remain; the same assay method can give different results in different laboratories, or in the same laboratory over time. Many medical practitioners have limited appreciation of preanalytic and analytic variability and of uncertainty of measurement. Closer liaison between pathology laboratories and referring clinicians will assist interpretation of test results and optimize outcomes for patients.

## Notes

***Disclosure Summary:*** The authors have nothing to disclose.

